# Blue Honeysuckle Extract Ameliorates Hyperuricemia by Modulating Gut Microbiota and Improving Liver–Kidney–Gut Axis Function

**DOI:** 10.3390/antiox15081015

**Published:** 2026-08-14

**Authors:** Xinyu Heng, Shaohong Huang, Qingyao Luo, Ke Zhang, Pei Liu, Yanan Zhu, Ruoxuan Deng, Ning Sun, Chen Qian, Chao Ye, Hai Yang

**Affiliations:** 1 Department of Central Laboratory, Xishan Hospital Affiliated to Jiangnan University (Preparatory), Jiangnan University, Wuxi 214105, China; 7222808014@stu.jiangnan.edu.cn; 2 MOE Medical Basic Research Innovation Center for Gut Microbiota and Chronic Diseases, Wuxi School of Medicine, Jiangnan University, Wuxi 214122, China; 6232803017@stu.jiangnan.edu.cn (S.H.); 1282230401@stu.jiangnan.edu.cn (Q.L.); 7232808009@stu.jiangnan.edu.cn (K.Z.); 6242803009@stu.jiangnan.edu.cn (P.L.); 7242808019@stu.jiangnan.edu.cn (Y.Z.); ruoxuan.deng@jiangnan.edu.cn (R.D.); sunning@jiangnan.edu.cn (N.S.); 3 Department of Clinical Laboratory, Xishan Hospital Affiliated to Jiangnan University (Preparatory), Jiangnan University, Wuxi 214105, China; qianchen@wxxsph.cn

**Keywords:** Hyperuricemia, blue honeysuckle, in vitro digestion, liver–kidney–gut axis, gut microbiota, untargeted metabolomics

## Abstract

Hyperuricemia (HUA) is a chronic metabolic disorder resulting from abnormal uric acid (UA) metabolism. Common clinical drugs for HUA have serious and inevitable adverse effects. Blue honeysuckle (*Lonicera caerulea* L.), a traditional edible berry with antioxidant and anti-inflammatory properties, shows great potential for alleviating HUA. This study prepared blue honeysuckle extract (BHE) and identified its main components as chlorogenic acid, quercetin, kaempferol, anthocyanin, etc., using LC-MS analysis. After in vitro digestion, BHE at concentrations ≥ 10 mg/mL retained over 70% of active components and biological activities. In HUA mice, BHE reduced serum UA levels, alleviated oxidative stress and inflammation, decreased UA production by inhibiting xanthine oxidase activity, and promoted renal and intestinal UA excretion through dual regulation of urate transporters. Additionally, BHE repaired the intestinal barrier, remodeled the gut microbiota structure, and improved UA metabolism disorders. This study supports BHE as a potential functional food ingredient for alleviating HUA.

## 1. Introduction

Hyperuricemia (HUA) is a chronic metabolic disorder characterized by excessive accumulation of serum uric acid (UA) due to abnormal purine metabolism or impaired UA excretion [1]. With dramatic changes in modern lifestyles, including high-purine diets, excessive alcohol consumption, and sedentary behavior, the prevalence of HUA continues to rise globally, establishing it as a major public health concern alongside obesity, diabetes, and hypertension [2]. HUA not only serves as the pathological basis of gout but is also closely associated with the development and progression of hypertension, chronic kidney disease, fatty liver, and other diseases [3]. Current clinical pharmacotherapy for HUA primarily includes xanthine oxidase (XOD) inhibitors (e.g., allopurinol, febuxostat) and uricosuric agents (e.g., benzbromarone) [4]. However, these drugs present significant limitations and adverse effects. Allopurinol may induce severe hypersensitivity reactions and hepatotoxicity [5]. Febuxostat has been linked to an increased risk of cardiovascular events [6]. Long-term administration of benzbromarone may lead to renal impairment [7]. Therefore, there is an urgent need to develop effective and low-toxicity interventions for HUA.

Compared with chemically synthesized drugs, food-derived active substances usually have higher biosafety and lower toxic side effects, making them ideal candidates for the adjuvant intervention and prevention of chronic metabolic diseases [8]. In addition to pharmacological therapy, dietary modification is an important component of HUA management. Recent evidence suggests that appropriately designed plant-based dietary patterns may contribute to improved UA homeostasis and may reduce the risk of kidney and cardiovascular complications. These findings support the investigation of food-derived bioactive ingredients as complementary strategies for HUA management [9]. Berries, in particular, have attracted considerable attention due to their outstanding contents of phenolics, flavonoids, and anthocyanins [10]. These active compounds possess strong antioxidant and anti-inflammatory capacities, showing great potential in the intervention of metabolic diseases. Blue honeysuckle (*Lonicera caerulea* L., BH), a deciduous shrub belonging to the Caprifoliaceae family, is widely distributed in cold regions such as Northeast China, Russia, and Canada [11]. Its berry is commonly processed into products such as juice, jam, and wine, contributing to its popularity among consumers worldwide. BH is rich in bioactive compounds, including phenolics, flavonoids, and anthocyanins, and exhibits multiple biological activities such as antioxidant, anti-inflammatory, anti-diabetic, and antihypertensive effects [12,13]. However, research on the ameliorative effect of BH on HUA remains scarce. Previous studies have found that the total phenol and total flavonoid contents of BH are significantly higher than those of common berries such as blueberries and mulberries [14], suggesting that it may have greater potential for improving HUA. Therefore, investigating the ameliorative effects and underlying mechanisms of BH on HUA is of great research value

UA is the end product of purine metabolism, and its homeostasis is tightly regulated by the liver–kidney–gut axis [15]. UA is primarily produced in the liver via XOD [16], while its excretion is mainly mediated by the kidneys (approximately 70%) and the intestines (approximately 30%) through urate transporters such as urate transporter 1 (URAT1), glucose transporter 9 (GLUT9), and ATP-binding cassette subfamily G member 2 (ABCG2) [17]. Dysregulation of hepatic XOD activity or impairment in UA reabsorption and excretion can result in elevated serum UA levels and the development of HUA [18]. Targeting the liver–kidney–gut axis to regulate UA metabolic homeostasis is therefore an effective strategy for HUA intervention. At present, various active components such as quercetin and anthocyanins have been identified in berries, which can improve HUA by mitigating liver–kidney–gut axis damage. Gut microbiota dysbiosis is closely associated with HUA, as it modulates intestinal barrier integrity (via tight junctions like Zonula occludens-1 (ZO-1)/Occludin), urate transporter expression, and purine degradation [19]. HUA is typically characterized by a reduction in beneficial bacteria (e.g., *Akkermansia*, *Lactobacillus*) and an increase in harmful bacteria (e.g., *Desulfovibrio*, *Escherichia coli*), which collectively lead to impaired intestinal UA excretion, disrupted intestinal barrier, and systemic inflammation [20]. Regulating gut microbiota balance has emerged as a novel and safe intervention strategy for HUA. Numerous phenolic and flavonoid compounds, as well as natural plants rich in these compounds (e.g., blueberries and mulberries), have been proven to be effective in ameliorating gut microbiota dysbiosis, further regulating metabolic homeostasis and improving HUA [21,22]. However, whether BH alleviates HUA by improving liver-kidney-intestinal axis function and gut microbiota dysbiosis has not yet been reported.

In this study, bule honeysuckle extract (BHE) was prepared and its main bioactive components were identified by liquid chromatography–tandem mass spectrometry (LC-MS/MS). Different concentrations of BHE were subjected to simulated gastrointestinal digestion to compare the changes in main bioactive components, XOD inhibitory activity, and radical scavenging activities after digestion. In animal experiments, BHE significantly reduced serum UA levels, alleviated oxidative stress and inflammation, decreased UA production by inhibiting XOD activity, promoted renal and intestinal UA excretion through regulation of urate transporters, and repaired the intestinal barrier by regulating tight junction protein expression. 16S rRNA gene sequencing of mouse cecal contents and serum untargeted metabolomics analysis revealed that BHE regulated gut microbiota and UA metabolism by enriching beneficial bacteria and suppressing harmful bacteria. This study explores the potential and mechanisms of BHE in ameliorating HUA and provides a scientific basis for the development of its related functional food ingredients, holding significant scientific and industrial value.

## 2. Materials and Methods

### 2.1. Materials and Reagents

α-Amylase, pepsin, gastric lipase, trypsin, bile salts, XOD, and xanthine were purchased from Sigma-Aldrich Trading Co., Ltd. (Shanghai, China). Potassium oxonate (PO, purity ≥ 99%), adenine (AD, purity ≥ 99%), and allopurinol (purity ≥ 99%) were obtained from Shanghai Aladdin Biochemical Technology Co., Ltd. (Shanghai, China). All antibodies used in this study were purchased from Proteintech Group (Wuhan, China). Other reagents were of analytical grade or higher and were purchased from Sinopharm Chemical Reagent Co., Ltd. (Shanghai, China).

### 2.2. Preparation of BHE and Chemical Characterization

BH (cultivar “Beilei”) was purchased from FengRan Agricultural Group Company (Jiamusi City, China). The extraction and purification of BHE were performed according to previously described methods with minor modifications [23]. Dried mature fruits of blue honeysuckle (*Lonicera caerulea* L.) were used to prepare the ethanol extract, hereafter referred to as blue honeysuckle extract (BHE), with 70% ethanol at a solid-to-liquid ratio of 1:10 (*w*/*v*). Each extraction was conducted at 65 °C for 1.5 h. The combined extracts were filtered and concentrated under reduced pressure at 60 °C to remove the organic solvent. The resulting concentrate was purified using an AB-8 macroporous resin column, which was washed with distilled water and subsequently eluted with 95% ethanol. The ethanol eluate was concentrated under reduced pressure at 55 °C and then freeze-dried to obtain the final BHE powder.

For LC-MS/MS analysis, 1 mg of BHE was dissolved in 1 mL of 20% methanol solution, and the mixture was centrifuged to collect the supernatant. Chromatographic separation was carried out on an Agilent 1290 Infinity II system (Santa Clara, CA, USA) equipped with a Waters ACQUITY UPLC HSS T3 column maintained at 35 °C. The mobile phase consisted of (A) acetonitrile and (B) 0.1% formic acid in water, at a flow rate of 0.3 mL/min. The gradient elution program was as follows: 0–30 min, 0% to 15% A; 30–45 min, 15% to 50% A; 45–55 min, 50% to 90% A; 55–60 min, 90% A; 60–60.1 min, 90% to 0% A; and 60.1–66 min, 0% A for column re-equilibration. The effluent was monitored using a diode array detector (DAD) at 254 nm. Mass spectrometric analysis was performed on an Agilent 6546 Q-TOF mass spectrometer (Santa Clara, CA, USA) equipped with an electrospray ionization (ESI) source operating in both positive and negative ion modes. Raw data were processed using Agilent MassHunter Qualitative Analysis 10.0 and SIRIUS 5.8.5 software.

### 2.3. In Vitro Digestion of BHE

Simulated in vitro digestion of BHE was carried out following the INFOGEST 2.0 protocol with slight modifications [24]. BHE was dissolved in distilled water to obtain concentrations of 1, 2.5, 5, 10, and 20 mg/mL. Each sample was mixed with simulated salivary fluid (1:1, *v*/*v*), gently agitated, and incubated at 37 °C for 2 min. Subsequently, the mixture was combined with simulated gastric fluid (1:1, *v*/*v*) and incubated at 37 °C for 2 h at pH 3.0. Following gastric digestion, the sample was mixed with simulated intestinal fluid (1:1, *v*/*v*) and incubated at 37 °C for 2 h at pH 7.0. After digestion, the samples were centrifuged at 1730× *g* for 15 min at 4 °C, and the resulting supernatants were stored at –80 °C for further analysis.

### 2.4. Determination of Total Phenol, Total Flavonoid, and Total Anthocyanin Contents

The total phenol, total flavonoid, and total anthocyanin contents in BHE were measured using commercial assay kits (Sangon Biotech, Shanghai, China), according to the manufacturer’s protocol. The total phenol content was calculated from the standard curve and expressed as μg gallic acid equivalents (GAE)/mg. The total flavonoid content was calculated from the standard curve and expressed as μg rutin equivalents (RE)/mg. The total anthocyanin content was calculated from the standard curve and expressed as μg cyanidin-3-glucoside equivalents (C3GE)/mg.

### 2.5. Determination of the XOD Inhibition Activity

The XOD inhibition activity of BHE was assessed using the previous methods with minor modifications [25]. In a 96-well microplate, a 50 μL sample, 60 μL 0.075 mol/L PBS buffer (pH 7.5), and 30 μL 0.1 U/mL XOD solution were sequentially added. The mixture was incubated at room temperature for 15 min. Then, 60 μL 0.05 mol/L xanthine substrate solution was added to initiate the reaction, which proceeded at room temperature for 5 min. The absorbance was measured at 295 nm using the EnSight^®^ Multimode Plate Reader (PerkinElmer, Waltham, MA, USA). The XOD inhibition rate was calculated using the following formula: XOD inhibition rate (%) = [A1 − (A2 − A3)]/A1 ∗ 100. In the formula, A1, A2, and A3 represent the absorbance changes in the blank control (without sample), the test sample, and the sample blank (without both sample and XOD), respectively.

### 2.6. Determination of DPPH and ABTS Free Radical Scavenging Activities

The DPPH and ABTS free radical scavenging activities of BHE were evaluated using commercial assay kits (Solarbio, Beijing, China) in accordance with the manufacturer’s protocol. The DPPH free radical scavenging rate was calculated as follows: DPPH free radical scavenging rate (%) = [(A0 − A1)/A0] ∗ 100, where A0 is the absorbance of the blank control and A1 is the absorbance of the sample group. The same formula was applied for ABTS free radical scavenging rate.

### 2.7. Animals and Experimental Design

Male C57BL/6J mice (8 weeks old) were obtained from GemPharmatech Co., Ltd. (Nanjing, China). All animals were housed in a specific pathogen-free facility under controlled environmental conditions (temperature: 22 ± 2 °C; humidity: 55 ± 5%; 12 h light/dark cycle) with free access to standard chow and sterilized water. All procedures involving animals were conducted in accordance with the Guidelines for the Care and Use of Laboratory Animals (NIH Publication, revised 2011) and were approved by the Animal Care and Use Committee of Jiangnan University (Approval No. JN.NO20260228C040035[035]).

After a 7-day acclimatization period, the mice were randomly divided into five groups (*n* = 8 per group): normal control (CON), HUA model (HUA), low-dose BHE (30 mg/kg, BHEL), high-dose BHE (150 mg/kg, BHEH), and positive control (5 mg/kg allopurinol, PC). The modeling of HUA and the dosage of drugs referred to the previous methods [15,26]. All reagents were dissolved in 0.5% sodium carboxymethyl cellulose (CMC-Na). Except for the CON group, mice in other groups were administered 250 mg/kg PO and 75 mg/kg AD daily by gavage, while the CON group received an equivalent volume of 0.5% CMC-Na. Mice in the BHEL, BHEH, and PC groups were respectively given low-dose BHE, high-dose BHE, and allopurinol daily by gavage, while the remaining groups received an equivalent volume of 0.5% CMC-Na. Body weight and average daily food intake were recorded weekly throughout the experimental period. After four weeks, the mice were fasted for 12 h and then anesthetized with isoflurane. Blood samples were collected from the orbital venous plexus and centrifuged at 3000× *g* for 15 min at 4 °C to obtain serum. The mice were subsequently euthanized by cervical dislocation, and the liver, kidney, colon tissues, and cecal contents were collected for further analysis.

### 2.8. Biochemical Assays

Serum levels of superoxide dismutase (SOD), catalase (CAT), malondialdehyde (MDA), interleukin-6 (IL-6), interleukin-1β (IL-1β), and tumor necrosis factor-α (TNF-α), as well as XOD activity in serum and liver, were measured using commercial assay kits (Elabscience, Wuhan, China) following the manufacturer’s instructions. Serum levels of UA, alanine aminotransferase (ALT), aspartate aminotransferase (AST), urea (UREA), and creatinine (CREA) were determined using an automatic biochemical analyzer (AU680, Beckman Coulter, Brea, CA, USA).

### 2.9. Histopathological Examination

Liver, kidney, and colon tissues were fixed in 4% paraformaldehyde for 24 h and then embedded in paraffin. Sections of 5 μm thickness were prepared and stained with hematoxylin and eosin (H&E) using a commercial kit (Beyotime, Shanghai, China) according to the manufacturer’s standard protocols.

### 2.10. Western Blot Analysis

Tissue samples were lysed in RIPA buffer supplemented with protease inhibitors. Protein concentrations were determined using a BCA protein assay kit (Thermo Fisher Scientific, Waltham, MA, USA). Equal amounts of protein were separated by SDS-PAGE and transferred onto PVDF membranes. Membranes were blocked with 5% skim milk for 1 h at room temperature and then incubated overnight at 4 °C with primary antibodies. After washing, membranes were incubated with HRP-conjugated secondary antibodies for 1 h at room temperature. Protein bands were visualized using an enhanced chemiluminescence reagent and detected with a Tanon imaging system (Shanghai, China).

### 2.11. RNA Extraction and Quantitative Real-Time PCR (qRT-PCR)

Total RNA was extracted from tissues using TRIzol reagent (Vazyme, Nanjing, China) according to the manufacturer’s protocol. RNA concentration and purity were assessed using a NanoDrop spectrophotometer (Thermo Fisher Scientific, Waltham, MA, USA). cDNA was synthesized using HiScript IV All-in-One Ultra RT SuperMix (Vazyme, Nanjing, China). The qRT-PCR was performed with gene-specific primers (listed in Table 1) using Gapdh as an internal reference gene. Relative gene expression levels were calculated using the 2^−ΔΔCt^ method.

### 2.12. Immunohistochemistry (IHC)

Paraffin-embedded tissue sections were deparaffinized in xylene and rehydrated through a graded ethanol series. Antigen retrieval was performed by boiling the sections in citrate buffer (pH 6.0) for 15 min. Endogenous peroxidase activity was blocked by incubating with 3% H_2_O_2_ for 10 min, followed by blocking with 5% bovine serum albumin for 1 h at room temperature. Sections were then incubated with primary antibodies overnight at 4 °C, followed by incubation with anti-mouse/rabbit EnVision™+/HRP reagent (Proteintech, Wuhan, China) for 1 h at 37 °C. Immunoreactivity was visualized using diaminobenzidine, and sections were examined under a light microscope (ECHO, San Diego, CA, USA).

### 2.13. 16S rRNA Sequencing of Gut Microbiota

Total genomic DNA was extracted from mouse cecal contents using the Magnetic Soil and Stool DNA Kit (Tiangen Biotech, Beijing, China) according to the manufacturer’s instructions. DNA concentration was measured using a Qubit fluorometer (Invitrogen, Carlsbad, CA, USA). The V3–V4 hypervariable region of the bacterial 16S rRNA gene was amplified using primers 341F (5′-CCTACGGGNGGCWGCAG-3′) and 805R (5′-GACTACHVGGGTATCTAATCC-3′). Sequencing was performed on the Illumina HiSeq platform by Lianchuan Biotechnology Co., Ltd. (Zhejiang, China). Bioinformatic analysis and visualization were conducted using OmicStudio tools available at https://www.omicstudio.cn/tool, accessed on 3 August 2026).

### 2.14. Serum Untargeted Metabolomics

Serum untargeted metabolomic analysis was carried out by Lianchuan Bioinformatics Co., Ltd. (Hangzhou, China). Briefly, 100 μL of serum was mixed with 400 μL of methanol, vortexed for 30 s, and incubated at −20 °C for 1 h to precipitate proteins. After centrifugation at 12,000× *g* for 15 min at 4 °C, the supernatant was collected and dried under a nitrogen stream. The residue was reconstituted in 100 μL of methanol–water (1:1, *v*/*v*), vortexed, and centrifuged again. The resulting supernatant was filtered through a 0.22 μm membrane and subjected to LC-MS/MS analysis. Bioinformatics analysis and integration with gut microbiota data were performed using OmicStudio tools at https://www.omicstudio.cn/tool, accessed on 3 August 2026).

### 2.15. Statistical Analysis

All data are presented as mean ± standard error of the mean (SEM) from at least three independent experiments. Statistical significance was assessed using one-way analysis of variance (ANOVA) with Tukey’s post hoc test. Image analysis was performed using ImageJ (version 1.54), and statistical graphs were generated using GraphPad Prism 10.0.2. *p* value < 0.05 was considered statistically significant.

## 3. Results

### 3.1. Preparation and Chemical Components Analysis of BHE

**Figure 1:**
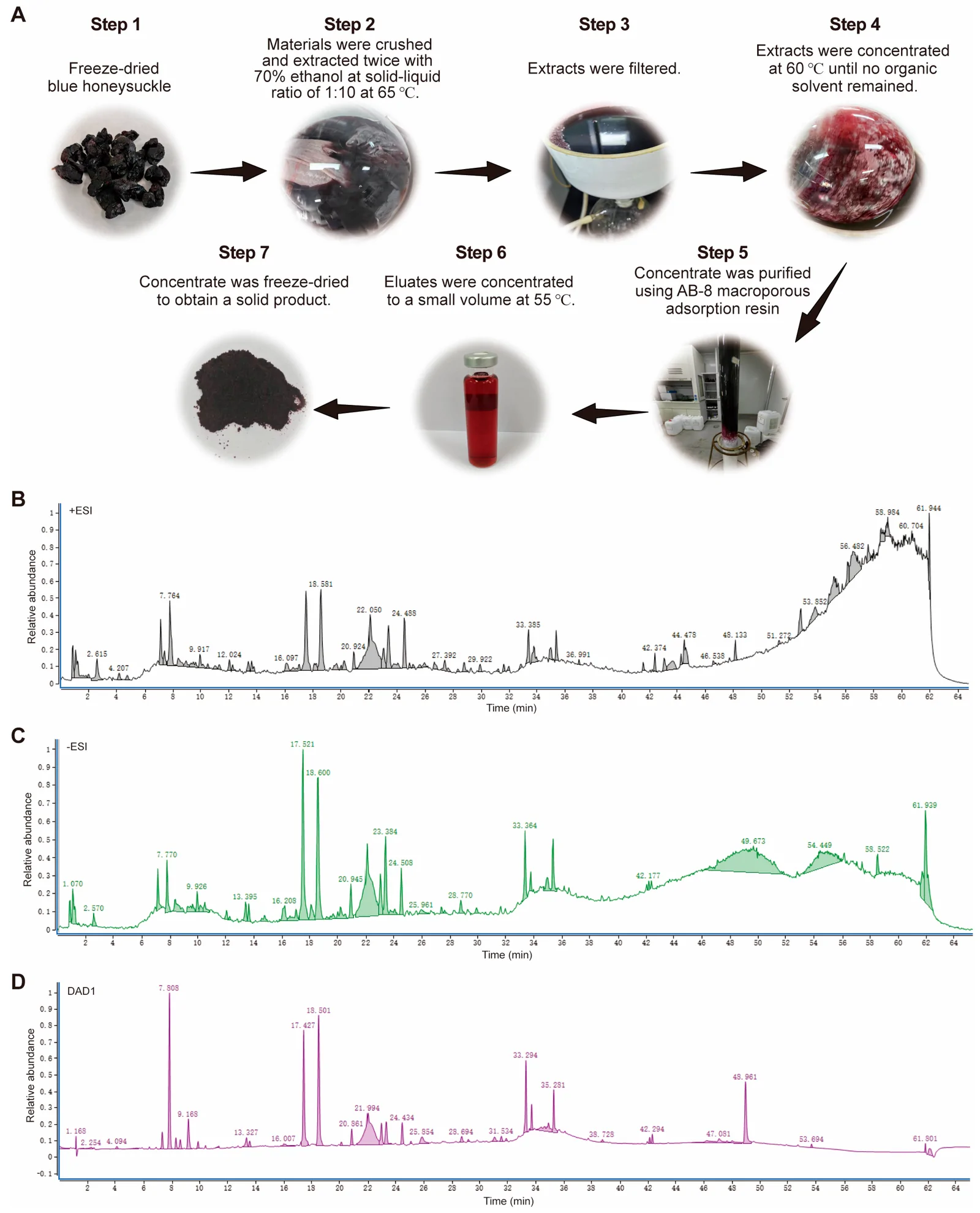
Preparation and chemical components analysis of BHE. (**A**) Schematic illustration of the extraction and purification process of BHE. (**B**–**D**) Representative LC-MS/MS and DAD chromatograms of BHE under ESI^+^ (**B**), ESI^−^ (**C**), and DAD (**D**) detection modes.

The dried BH underwent extraction and purification to yield the ethanol extract (BHE), following the protocol outlined in Figure 1A. The chemical components of BHE were characterized using LC-MS/MS, with detection performed in both positive and negative electrospray ionization (ESI^+^/ESI^−^) modes, coupled with diode array detection (DAD) (Figure 1B–D). Data processing and compound matching were conducted using Agilent Qualitative Analysis 10.0 software, along with the PCDL MS/MS database and the SIRIUS online database (v5.8.5). As presented in Table 2, BHE primarily contained phenolic acids, flavonoids, anthocyanins, and iridoids. Notably, chlorogenic acid derivatives (including chlorogenic acid, neochlorogenic acid, and cryptochlorogenic acid) and their dimers (e.g., 1,5-dicaffeoylquinic acid) exhibited strong signals in both ionization modes, suggesting these compounds are major phenolic acid components in BHE. Flavonoids were particularly abundant, comprising monoglycosides, diglycosides, and triglycosides of quercetin, kaempferol, and luteolin, such as rutin, hyperoside, and isoquercitrin. Additionally, anthocyanins like cyanidin-3-O-glucoside, as well as iridoid components including morronisic acid and morroniside, were also detected.

### 3.2. Effects of In Vitro Digestion on the Chemical Components and Bioactivities of BHE

**Figure 2:**
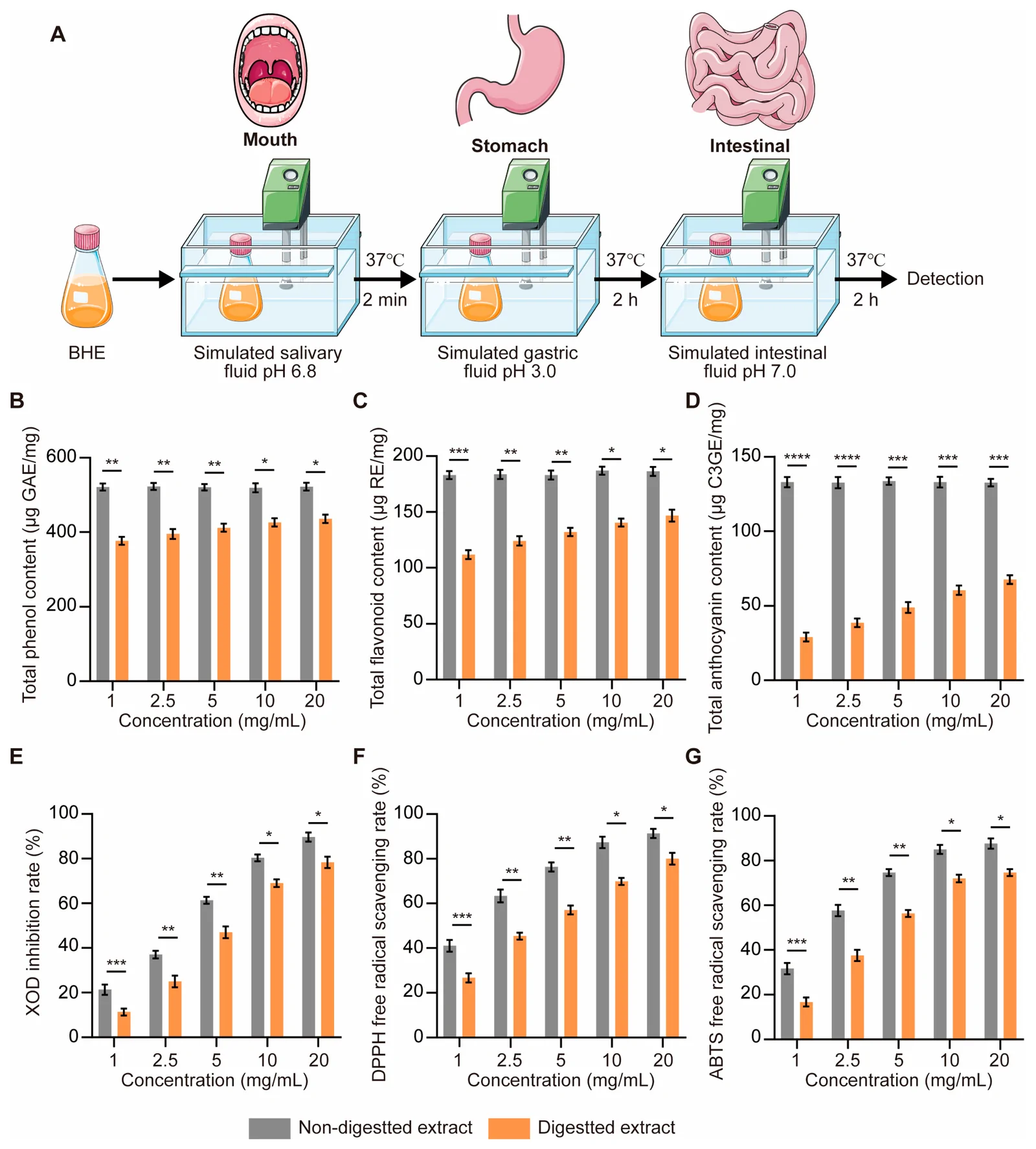
Effects of in vitro digestion on the chemical composition and activities of BHE. (**A**) Schematic of the in vitro digestion model. (**B**–**D**) Contents of total phenols (**B**), total flavonoids (**C**), and total anthocyanins (**D**) in undigested and digested BHE at different concentrations (1, 2.5, 5, 10, 20 mg/mL). (**E**–**G**) XOD inhibition rate (**E**), DPPH free radical scavenging rate (**F**), and ABTS free radical scavenging rate (**G**) of undigested and digested BHE at different concentrations (1, 2.5, 5, 10, 20 mg/mL). Data: mean ± SEM. * *p* < 0.05, ** *p* < 0.01, *** *p* < 0.001, **** *p* < 0.0001. Statistical significance was estimated by one-way analysis of variance (ANOVA) with Tukey’s post hoc test.

The changes in the components and activities of BHE during gastrointestinal transit were investigated using a continuous in vitro oral-gastric-intestinal digestion model (Figure 2A). Between undigested and digested BHE, the main chemical component contents and bioactivities were systematically compared. Given that BHE was predominantly composed of phenolics, flavonoids, and anthocyanins, the total contents of these three compound classes were selected as indicators. The results demonstrated that following digestion, the total phenolic, total flavonoid, and total anthocyanin contents were significantly lower than those in undigested BHE across various concentrations (Figure 2B–D). Among these, total anthocyanins exhibited the most pronounced decrease, indicating their relatively lower stability under digestive conditions. Although total phenolics and total flavonoids also decreased, over 70% of their contents were retained at higher concentrations (above 10 mg/mL).

Given that inhibiting XOD activity is a key therapeutic strategy for alleviating HUA, this study evaluated the XOD inhibition rate of BHE at different concentrations before and after digestion. Concurrently, as antioxidant activity is a fundamental biological property of plant extracts and its alteration directly reflects the impact of digestion on extract functionality, the DPPH and ABTS radical scavenging capacities of BHE were assessed in parallel. Bioactivity analysis revealed that undigested BHE significantly inhibited XOD activity and scavenged DPPH and ABTS radicals, demonstrating promising anti-hyperuricemic and antioxidant potential. Following digestion, the XOD inhibition rate and the DPPH and ABTS radical scavenging rates of BHE at each concentration were significantly lower than those of the undigested extract (Figure 2E–G). This decline in bioactivity generally coincided with the reduction in bioactive component contents. Notably, at concentrations of 10 mg/mL and above, the digested BHE still retained over 70% radical scavenging capacity and XOD inhibition. These findings indicate that BHE undergoes partial degradation or structural transformation of its active components during simulated digestion. However, when consumed at concentrations of 10 mg/mL or higher, the core biological activities of BHE can be largely preserved.

### 3.3. Effects of BHE on Body Weight, Food Intake, and Serum Biochemical Indicators in HUA Mice

**Figure 3:**
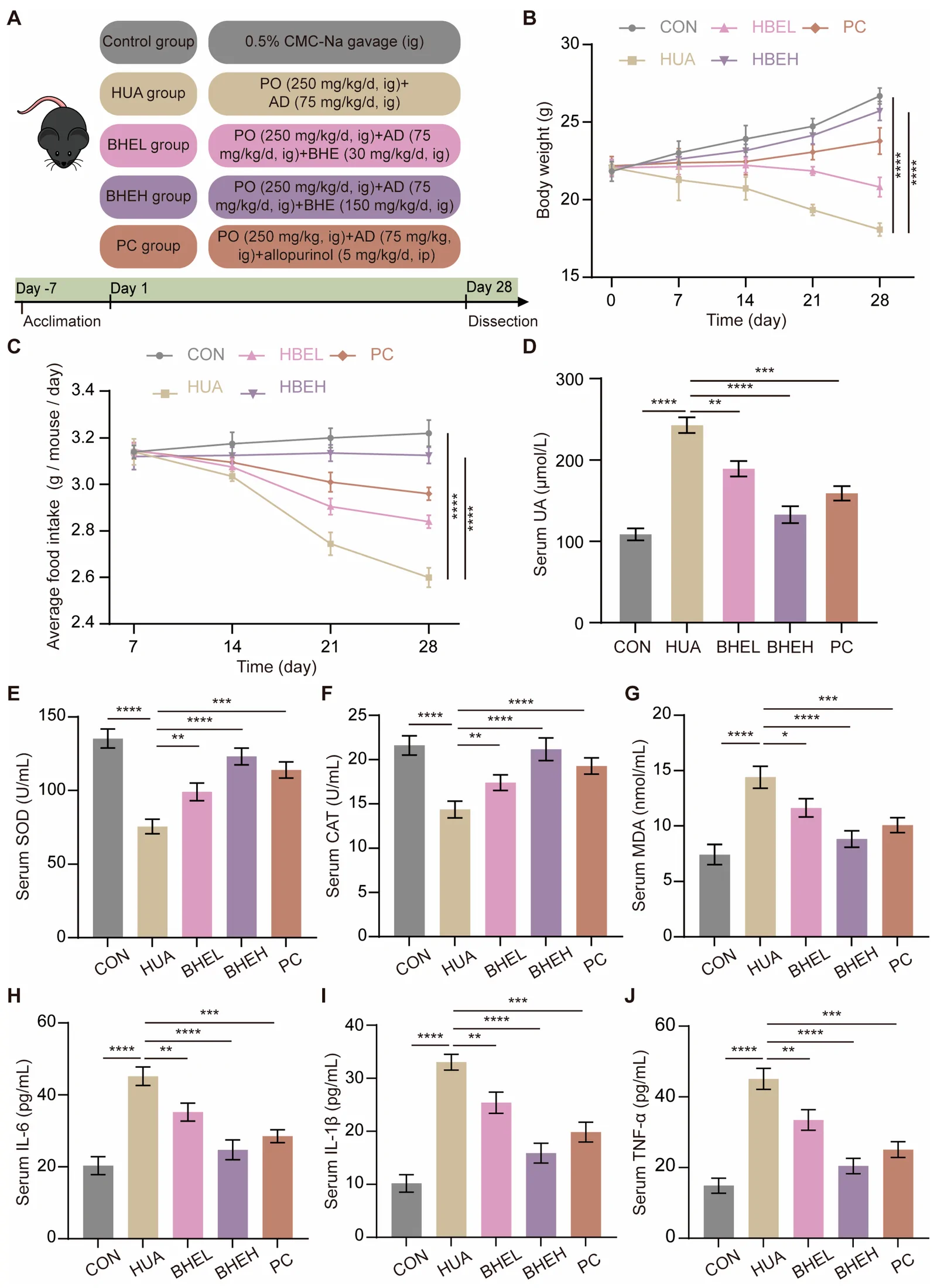
Effects of BHE on body weight, food intake, and serum biochemical indicators in HUA mice. (**A**) Schematic diagram of experimental animal grouping and intervention protocol. (**B**) Mouse body weight change. (**C**) Daily average food intake of mice. (**D**) Serum UA level in mice. (**E**–**G**) Serum SOD activity, catalase CAT activity, and MDA level in mice. (**H**–**J**) Serum IL-6, IL-1β, and TNF-α levels in mice. Data: mean ± SEM. * *p* < 0.05, ** *p* < 0.01, *** *p* < 0.001, **** *p* < 0.0001. Statistical significance was estimated by one-way analysis of variance (ANOVA) with Tukey’s post hoc test.

To evaluate the anti-hyperuricemic effect of BHE, a mouse model of HUA was established by intragastric administration of PO combined with AD. A combined PO/adenine model was used to induce elevated serum UA together with renal dysfunction. PO increases UA by inhibiting uricase, whereas adenine can be metabolized to poorly soluble 2,8-DHA and thereby contribute independently to tubular crystal formation and nephropathy. Thus, this model represents a mixed hyperuricemic and adenine-associated renal injury phenotype. BHE were administered as interventions, with allopurinol serving as the positive control (Figure 3A). Throughout the experiment, body weight and average daily food intake in the HUA group showed a significant decreasing trend, suggesting that a high UA load may lead to reduced appetite or metabolic disturbances. Following BHE intervention, both body weight and average daily food intake were markedly improved, with the recovery effect in the BHEH group surpassing that of the PC group (Figure 3B,C). At the end of the intervention, the serum UA concentration was approximately 100 μmol/L in the CON group and 230 μmol/L in the HUA group. Treatment with BHE and PC reduced serum UA to 180 μmol/L, 120 μmol/L, and 150 μmol/L in the BHEL, BHEH, and PC groups, respectively (Figure 3D). Concurrently, the activities of antioxidant enzymes such as SOD and CAT decreased significantly, while the levels of MDA and pro-inflammatory factors (IL-6, IL-1β, TNF-α) increased significantly (Figure 3E–J). This indicates that oxidative stress and inflammatory responses play important roles in the pathological process of HUA. After BHE intervention, these indicators exhibited a dose-dependent reversal trend. The ameliorative effect of BHEH was more pronounced and outperformed that of the PC group. These results demonstrate that BHE can effectively alleviate elevated serum UA levels, oxidative stress damage, and inflammatory responses in HUA mice.

### 3.4. Protective Effects of BHE on Liver and Kidney Functions and Histopathological Damage in HUA Mice

**Figure 4:**
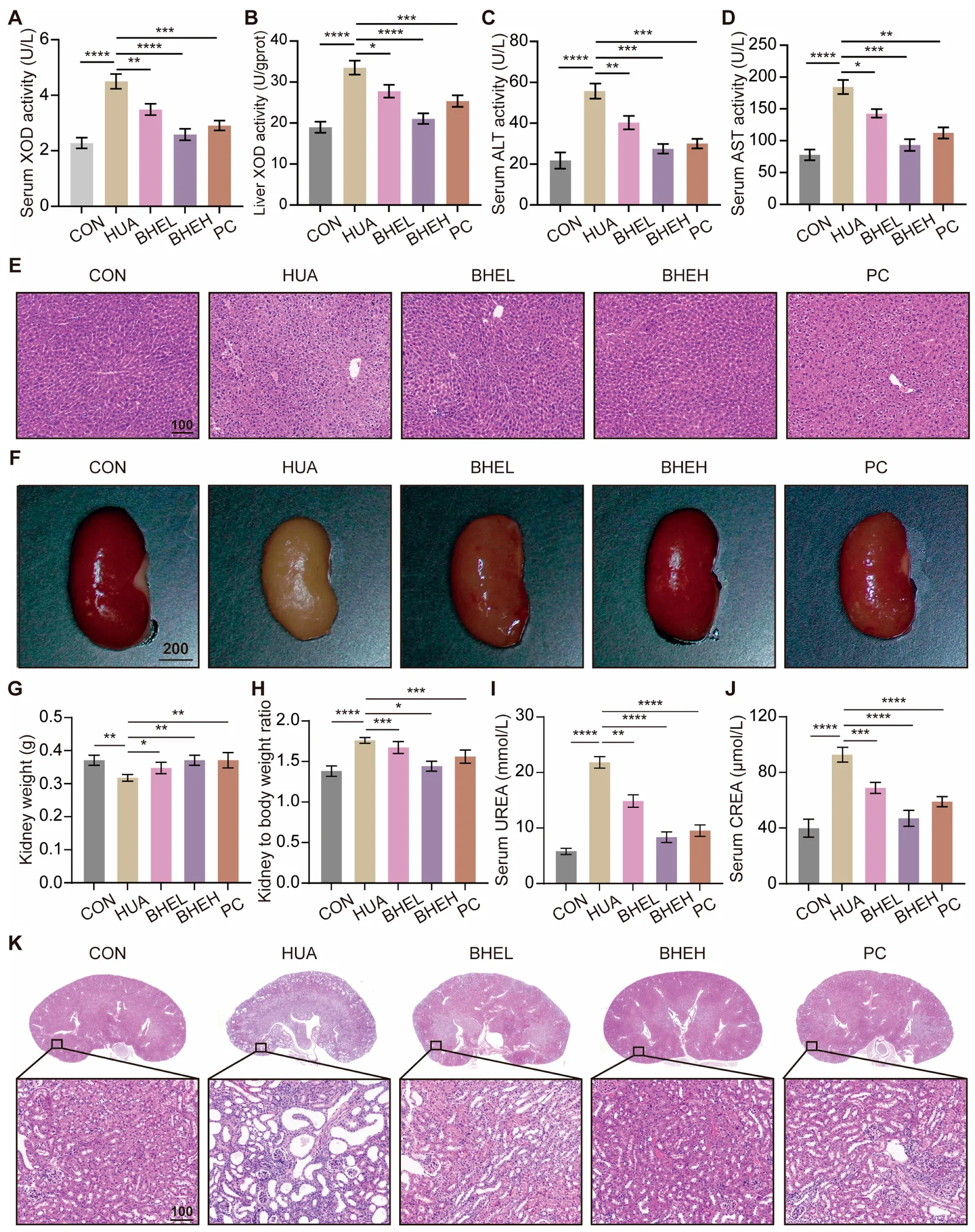
Protective effects of BHE on liver and kidney functions and histopathological damage in HUA mice. (**A**) Serum XOD activity in mice. (**B**) Liver XOD activity in mice. (**C**,**D**) Serum ALT and AST activities in mice. (**E**) H&E staining of liver tissue (scale bar = 100 μm). (**F**) Gross morphology observation of the kidney (scale bar = 200 μm,). (**G**,**H**) Mouse kidney weight and kidney-to-body weight ratio. (**I**,**J**) Serum UREA and CREA levels in mice. (**K**) H&E staining of kidney tissue (scale bar = 100 μm). Data: mean ± SEM. * *p* < 0.05, ** *p* < 0.01, *** *p* < 0.001, **** *p* < 0.0001. Statistical significance was estimated by one-way analysis of variance (ANOVA) with Tukey’s post hoc test.

The liver serves as the central organ for UA synthesis, and abnormal purine metabolism mediated by XOD is a key driver of HUA [27]. As shown in Figure 4A,B, compared with the CON group, serum and hepatic XOD activities were significantly elevated in the HUA group, indicating an abnormal enhancement in the activity of key hepatic UA-producing enzymes under high UA load. Concurrently, serum levels of ALT and AST were significantly increased (Figure 4C,D), suggesting impaired hepatocyte function. H&E staining of liver tissues further confirmed these findings, revealing marked pathological changes in the HUA group (Figure 4E), including hepatocellular swelling, cytoplasmic rarefaction, inflammatory cell infiltration, and disorganized hepatic cord arrangement. Following BHE intervention, serum and hepatic XOD activities decreased in a dose-dependent manner. ALT and AST levels also significantly declined. The pathological structure of the liver was notably improved, with reduced hepatocellular swelling and inflammatory infiltration, and hepatic cords appearing more orderly. The protective effect of BHE was comparable to that of the PC group.

The kidneys are the primary route for UA excretion, through filtration and reabsorption [28]. Renal impairment not only exacerbates UA accumulation but can also cause second kidney damage due to urate deposition. As shown in Figure 4F, the kidneys of HUA mice appeared pale in gross morphology. The kidney-to-body weight ratio was significantly increased (Figure 4G,H), indicating renal edema and compensatory enlargement. Concurrently, serum levels of UREA and CREA were markedly elevated (Figure 4I,J), suggesting impaired renal function. H&E staining of renal tissues revealed typical pathological injuries, including renal tubular dilation, epithelial cell detachment, and inflammatory cell infiltration (Figure 4K). These changes are likely closely associated with tubular damage and excretory dysfunction triggered by urate deposition. After BHE intervention, the gross morphology and weight of the kidneys were significantly improved. Serum UREA and CREA levels were notably reduced. Renal pathological damage was alleviated in a dose-dependent manner. The protective effect was most prominent in the BHEH group, surpassing that of the PC group.

### 3.5. BHE Modulates the Expression Balance of Renal Urate Transporters in HUA Mice

**Figure 5:**
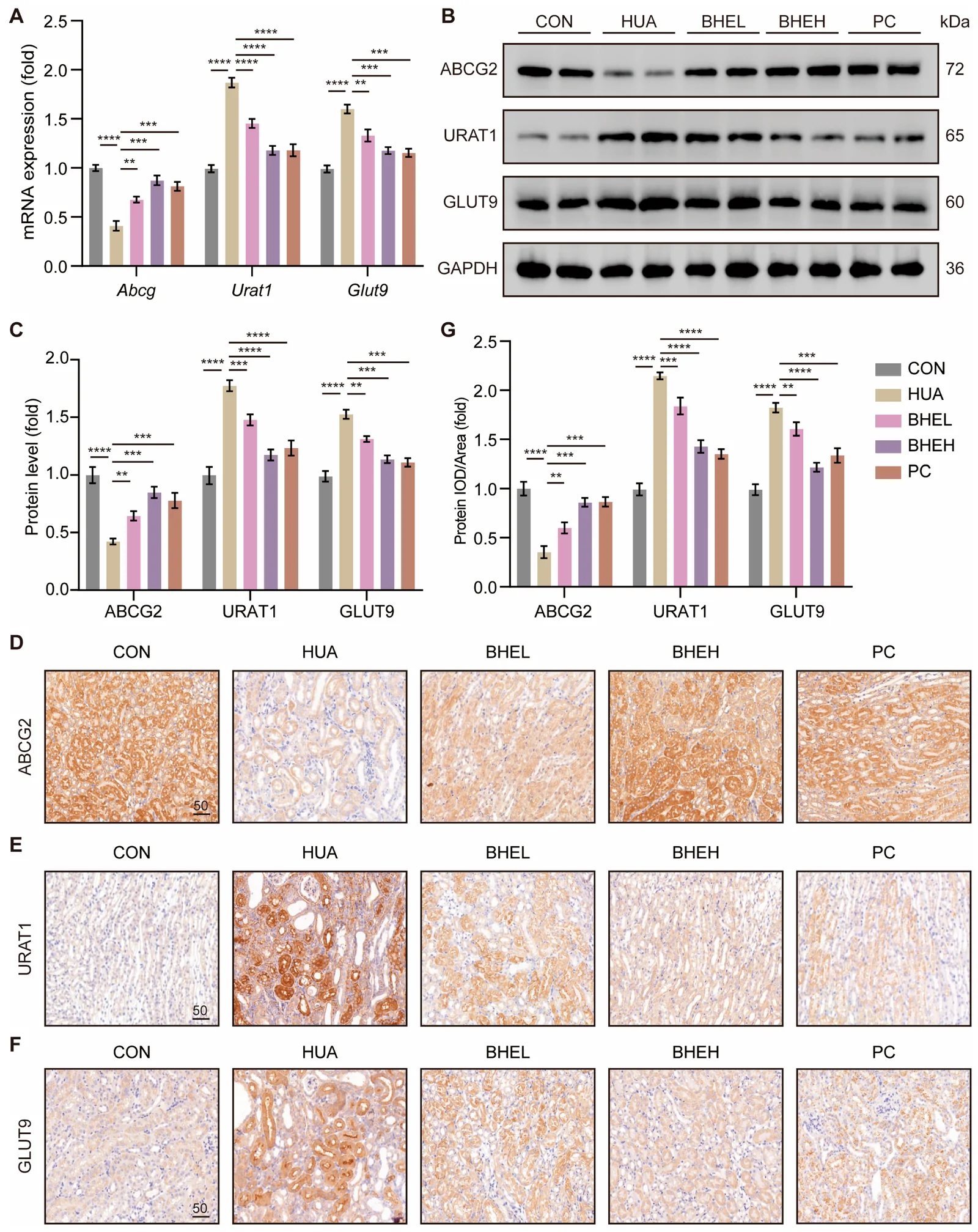
Regulatory effects of BHE on renal urate transporter expression in HUA mice. (**A**) mRNA expression levels of renal ABCG2, URAT1, and GLUT9. (**B**,**C**) Representative Western blots (**B**) and quantification (**C**) of ABCG2, URAT1, GLUT9, and GAPDH. (**D**–**G**) Representative ABCG2 (**D**), URAT1 (**E**), and GLUT9 (**F**) IHC images and quantification (**G**) (scale bars = 50 µm). Data: mean ± SEM. ** *p* < 0.01, *** *p* < 0.001, **** *p* < 0.0001. Statistical significance was estimated by one-way analysis of variance (ANOVA) with Tukey’s post hoc test.

The renal urate transporter system is central to regulating UA excretion and reabsorption [29]. Among these transporters, ABCG2 mediates urate excretion, while URAT1 and GLUT9 are responsible for urate reabsorption. An imbalance in their expression is a key mechanism in the development of HUA. The qRT-PCR and Western blot analysis revealed that compared with the CON group, both mRNA and protein expression levels of ABCG2 in the kidneys of HUA mice were significantly down-regulated (Figure 5A–C). In contrast, the mRNA and protein expression of URAT1 and GLUT9 were markedly up-regulated. These findings indicate that under HUA conditions, renal urate excretion is significantly impaired while reabsorption is abnormally enhanced. After BHE intervention, ABCG2 expression showed a dose-dependent recovery, while the expression of URAT1 and GLUT9 was significantly reduced. The regulatory effect of the BHEH group was comparable to that of the PC group. IHC staining further visually confirmed these results. In the HUA group, the positive staining intensity of ABCG2 was significantly weakened, primarily localized on the membrane and in the cytoplasm of renal tubular epithelial cells, with a notably reduced proportion of positive cells (Figure 5D). Conversely, positive staining for URAT1 and GLUT9 was significantly enhanced, widely distributed along the brush border and in the cytoplasm of renal tubular epithelial cells (Figure 5E–G), suggesting abnormally active reabsorption function. Following BHE intervention, the positive staining intensity and proportion of ABCG2 increased markedly, whereas the positive staining for URAT1 and GLUT9 was significantly attenuated. These results collectively demonstrate that BHE can effectively modulate the expression balance of renal urate transporters, thereby helping to restore the physiological homeostasis of UA excretion and reabsorption.

### 3.6. BHE Improves Intestinal Barrier Integrity and Modulates Intestinal Urate Transporter ABCG2 Expression in HUA Mice

**Figure 6:**
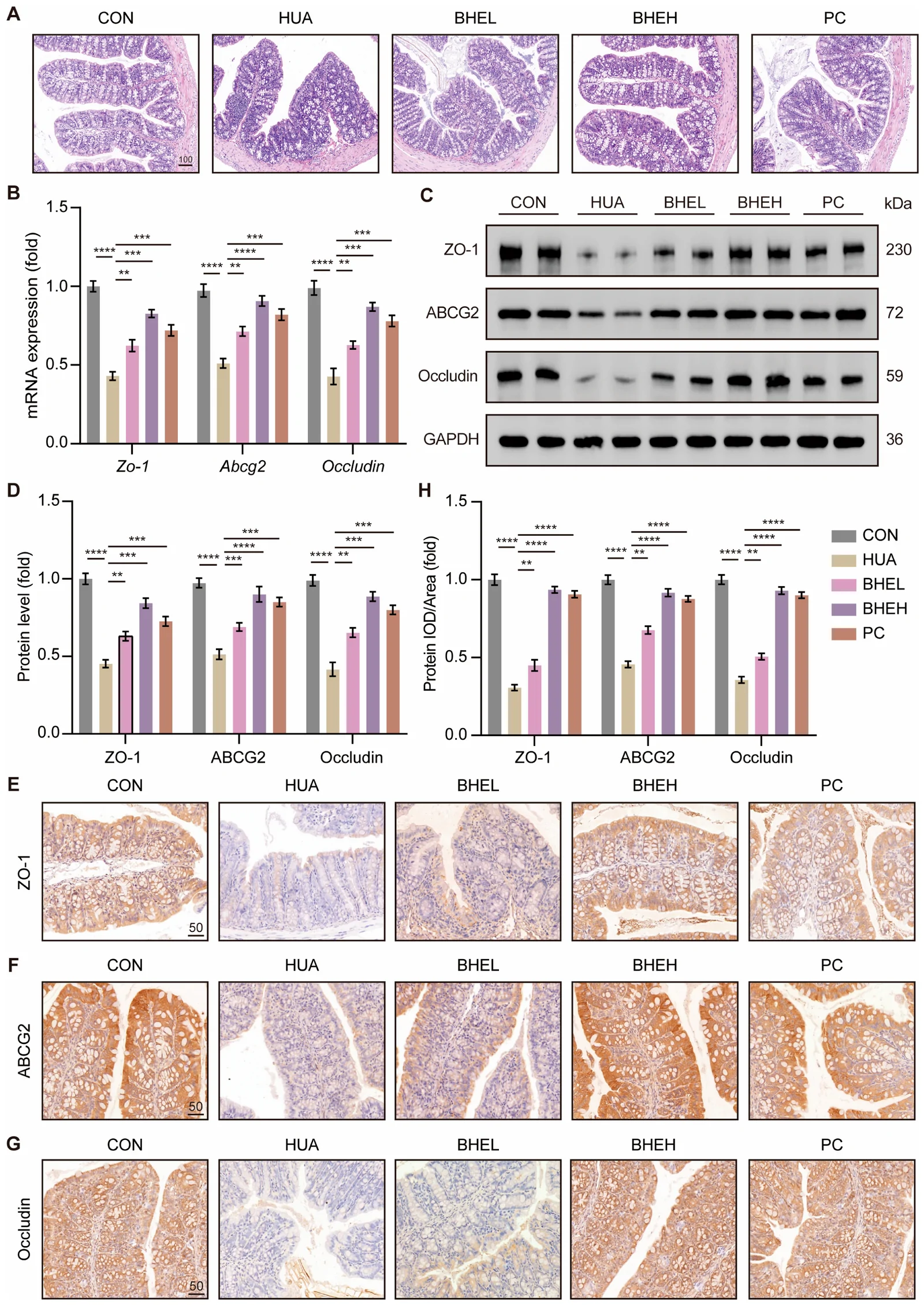
Effects of BHE on intestinal barrier function and intestinal urate transporter ABCG2 expression in HUA mice. (**A**) Representative H&E staining of intestinal sections (scale bar = 100 μm). (**B**) Relative mRNA expression levels of ZO-1, ABCG2, and Occludin in intestinal tissues. (**C**,**D**) Representative Western blots (**C**) and quantification (**D**) of ZO-1, ABCG2, and Occludin. (**E**–**H**) Representative ZO-1 (**E**), ABCG2 (**F**), and Occludin (**G**) IHC images and quantification (**H**) (scale bars = 50 µm). Data: mean ± SEM. ** *p* < 0.01, *** *p* < 0.001, **** *p* < 0.0001. Statistical significance was estimated by one-way analysis of variance (ANOVA) with Tukey’s post hoc test.

Intestinal barrier integrity and the function of the intestinal urate exporter ABCG2 are key aspects of the gut’s role in regulating UA metabolism [30]. H&E staining analysis demonstrated that compared with the CON group, the HUA group exhibited significant intestinal barrier damage, characterized by villus atrophy, crypt hyperplasia, and increased inflammatory cell infiltration (Figure 6A). After BHE intervention, the intestinal mucosal structure improved in a dose-dependent manner. The BHEH group displayed villus morphology close to normal and a significant reduction in the inflammatory response. The qRT-PCR and Western blot analysis indicated that compared with the CON group, the HUA group showed significant decreases in the mRNA and protein expression of ABCG2 and the tight junction proteins (ZO-1 and Occludin) (Figure 6B–D). BHE treatment reversed these changes in a dose-dependent manner, reaching levels comparable to the PC group. IHC results further visually substantiated these findings. In the HUA group, the positive staining intensity of ABCG2, ZO-1, and Occludin was significantly weakened, mainly distributed on the membrane and in the cytoplasm of intestinal epithelial cells (Figure 6E–H). After BHE intervention, the positive staining intensity and proportion of all three markers notably increased. These results demonstrate that BHE can improve intestinal barrier function and enhance intestinal urate excretion capacity by restoring the expression of intestinal tight junction proteins and the urate exporter in HUA mice.

### 3.7. BHE Remodels Gut Microbiota Composition and Function in HUA Mice

**Figure 7:**
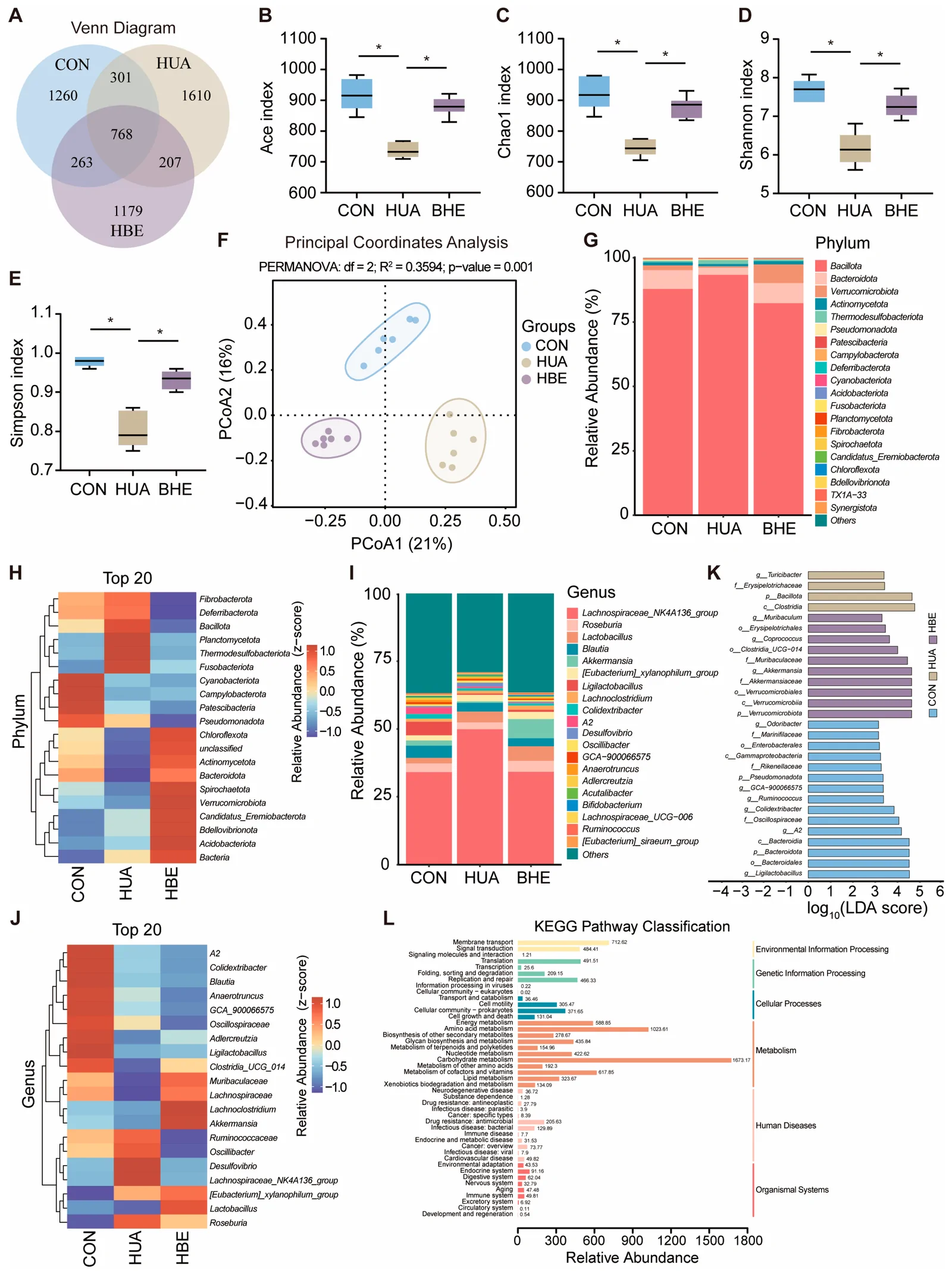
Regulatory effects of BHE on gut microbiota composition and function in HUA mice. (**A**) Venn diagram of OTUs from the gut microbiota of the three groups. (**B**–**E**) Gut microbiota α-diversity indices: Ace (**B**), Chao1 (**C**), Shannon (**D**), Simpson (**E**). (**F**) Principal coordinate analysis of gut microbiota. (**G**) Bar chart of relative abundance of gut microbiota at the phylum level. (**H**) Heatmap of relative abundance of gut microbiota at the phylum level. (**I**) Bar chart of relative abundance of gut microbiota at the genus level. (**J**) Heatmap of relative abundance of gut microbiota at the genus level. (**K**) Differential species identified by LEfSe analysis among groups (LDA threshold > 4). (**L**) KEGG enrichment analysis showing functional potential of gut microbiota. Data: mean ± SEM. * *p* < 0.05. Statistical significance was estimated by one-way analysis of variance (ANOVA) with Tukey’s post hoc test.

The gut microbiota participates in HUA progression by modulating UA metabolism, and its dysbiosis is a significant contributing factor in the development of HUA [31]. This study analyzed the gut microbiota characteristics of each group via 16S rRNA sequencing of mouse cecal contents. Venn diagram analysis revealed that the three groups shared 768 core operational taxonomic units (OTUs). The CON, HUA, and BHE groups possessed 1260, 1610, and 1179 unique OTUs, respectively, indicating significant alterations in microbial species composition under HUA conditions (Figure 7A). Alpha diversity analysis demonstrated that compared with the CON group, the HUA group exhibited significantly lower Ace, Chao1, and Shannon indices, and a significantly higher Simpson index (Figure 7B–E), suggesting impaired richness and diversity of the gut microbiota in HUA mice. BHE intervention significantly restored the Ace, Chao1, and Shannon indices and decreased the Simpson index, effectively recovering microbial diversity. Principal coordinate analysis demonstrated distinct separation among the three groups. The HUA group clustered apart from the CON group, while the microbiota structure of the BHE group was closer to that of the CON group, indicating that BHE significantly reshaped the gut microbiota composition in HUA mice (Figure 7F).

At the phylum level, Bacillota and Bacteroidota were the dominant phyla in all three groups. The relative abundances of Fibrobacterota, Deferribacterota, Bacillota, Planctomycetota, Thermodesulfobacteriota, and Fusobacteriota increased in the HUA group compared with the CON group, whereas the abundances of Actinomycetota, Bacteroidota, and Chloroflexota decreased (Figure 7G,H). BHE intervention reversed this trend and normalized the Bacillota to Bacteroidota ratio. At the genus level, compared with the CON group, the relative abundances of genera such as *Desulfovibrio* and *Oscillibacter* were significantly increased in the HUA group, while the relative abundances of genera such as *Clostridia_UCG-014* and *Lachnospiraceae* were significantly decreased. BHE intervention significantly increased the abundances of beneficial genera such as *Lachnospiraceae*, *Akkermansia*, and *Lactobacillus*, and reduced the abundance of potentially harmful genera such as *Desulfovibrio* (Figure 7I,J). Linear discriminant analysis Effect Size (LEfSe) analysis further identified key differential taxa among the groups (Figure 7K). Kyoto Encyclopedia of Genes and Genomes (KEGG) enrichment analysis based on 16S rRNA data revealed that, compared with the HUA group, the relative abundances of pathways related to carbohydrate metabolism, membrane transport, and amino acid metabolism were significantly increased in the BHE group (Figure 7L). Collectively, these results demonstrate that BHE can alleviate gut microbial dysbiosis in HUA mice by remodeling the gut microbiota structure and restoring microbial diversity.

### 3.8. BHE Improves UA Metabolic Disorders in HUA Mice

**Figure 8:**
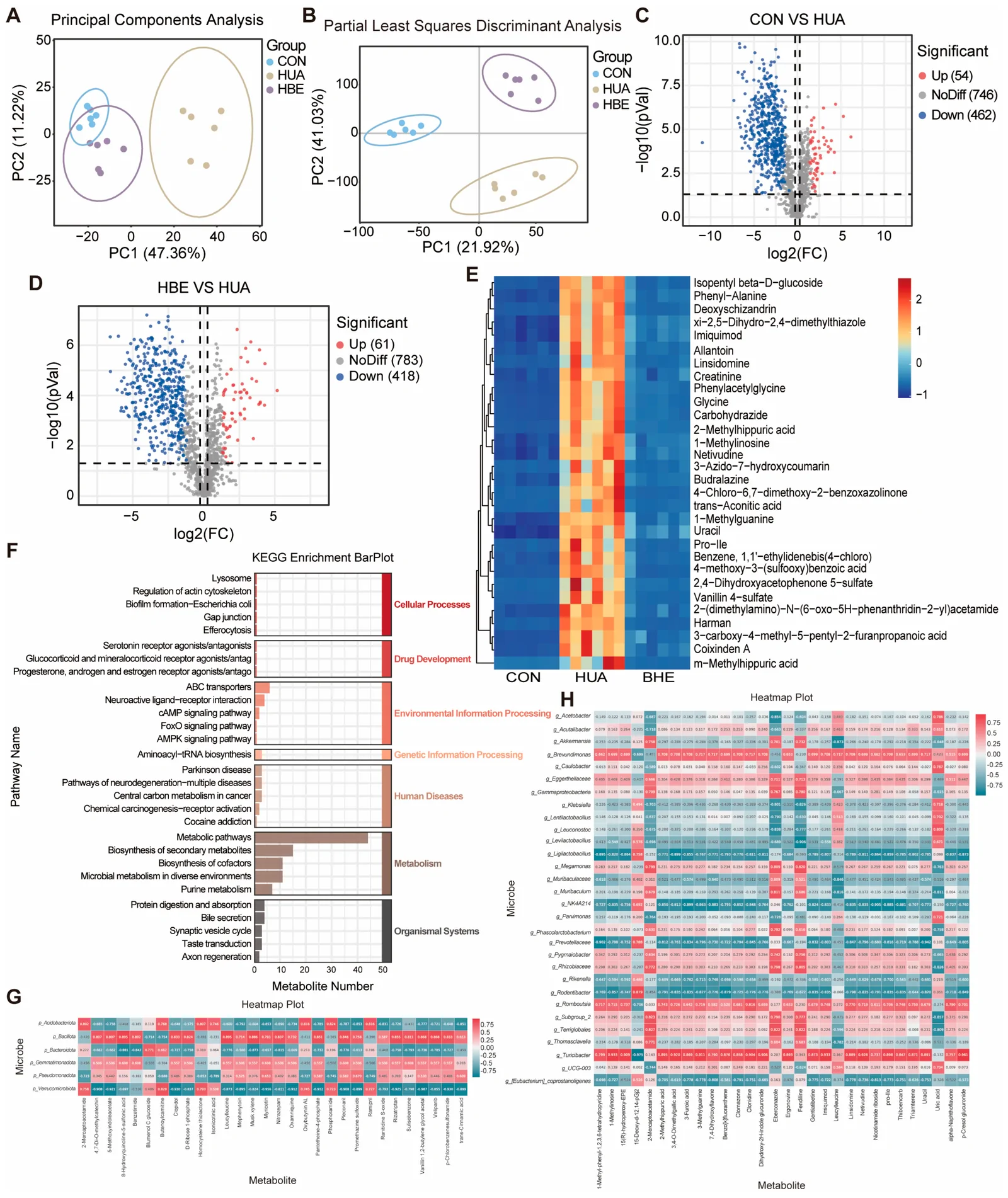
Regulatory effects of BHE on UA metabolic disorders in HUA mice. (**A**) Principal component analysis of serum metabolic profiles. (**B**) Partial least squares–discriminant analysis of serum metabolic profiles. (**C**) Volcano plot of differential serum metabolites between the CON and HUA groups. (**D**) Volcano plot of differential serum metabolites between the HBE and HUA groups. (**E**) Heatmap of top differential serum metabolites. (**F**) KEGG enrichment analysis of differential serum metabolites. (**G**,**H**) Heatmap of associations between differential serum metabolites and gut microbiota (phylum G and genus H). Data: mean ± SEM. * *p* < 0.05. Statistical significance was estimated by one-way analysis of variance (ANOVA) with Tukey’s post hoc test.

To elucidate the metabolic regulatory mechanism by which BHE ameliorates HUA, untargeted metabolomics was employed to analyze the serum metabolic profiles of mice. Principal component analysis and partial least squares–discriminant analysis revealed a clear separation in the serum metabolic profiles among the three groups. The HUA group exhibited a distinct metabolic signature compared to the CON group, whereas the metabolic profile of the BHE group shifted closer to that of the CON group (Figure 8A,B), suggesting that BHE significantly reversed the metabolic disturbances in HUA mice. Volcano plot analysis identified 514 differential metabolites (54 up-regulated, 462 down-regulated) between the CON and HUA groups, and 479 differential metabolites (61 up-regulated, 418 down-regulated) between the BHE and HUA groups (Figure 8C,D). Heatmap analysis of these differential metabolites demonstrated that metabolites related to UA metabolism, such as uracil, allantoin, creatinine, and phenylalanine, were significantly increased in the HUA group compared to the CON group (Figure 8E). BHE intervention significantly lowered the levels of these metabolites, indicating a marked improvement in UA metabolism.

KEGG enrichment analysis revealed that the differential metabolites were primarily enriched in pathways including purine metabolism, biosynthesis of secondary metabolites, and microbial metabolism in diverse environments (Figure 8F). Several signaling pathways, such as cAMP, AMPK, and FoxO, were also implicated. Correlation analysis was further conducted by integrating the gut microbiota data. A metabolite-gut microbiota correlation heatmap demonstrated significant associations between differential metabolites and gut microbiota. The phylum Bacillota correlated positively with metabolites such as 8-Hydroxyquinoline-5-sulfonic acid, leucylleucine, and 5-Methoxyindoleacetate, whereas Bacteroidota correlated negatively with these metabolites (Figure 8G). Beneficial genera like *Turicibacter*, *Akkermansia*, and *Lactobacillus* exhibited significant positive correlations with leucylleucine, 2-mercaptoacetamide, and 1-methylinosine, but negative correlations with metabolites such as uracil and UA (Figure 8H). Taken together, these results indicate that BHE can improve metabolic disorders in HUA mice through the regulation of gut microbiota.

## 4. Discussion

HUA has emerged as a prevalent metabolic disorder worldwide, with its incidence continuously rising due to changes in dietary patterns and lifestyles [32]. Chronic high UA load promotes the development of conditions such as gout, atherosclerosis, and hypertension [33]. Current pharmacological treatment options, primarily XOD inhibitors and uricosuric agents, are limited by their single-target mechanisms and substantial adverse effects, which include allergic reactions and renal injury [34]. Therefore, the development of low-toxicity and efficient interventions for HUA is urgently needed. BH, a medicinal and edible berry, is rich in various natural active components such as phenolic acids and flavonoids [35]. With its long history of consumption, low toxicity, and easy accessibility, it possesses inherent advantages for developing a functional food ingredient to intervene in HUA. This study systematically investigated the potential of BHE to ameliorate HUA through in vitro digestion and in vivo animal experiments. The optimal intake concentration of BHE was determined, and its underlying mechanisms were elucidated from multiple dimensions, including UA production, UA excretion, gut microbiota, and UA metabolism. These results provide theoretical support for the application of BHE as a functional food ingredient in the treatment and prevention of HUA.

BH is a traditional berry for daily consumption, and its extract retains the natural properties of food, avoiding the toxic side effects of chemical drugs, thus granting it unique natural advantages for long-term intervention in HUA. Previous studies have shown that the contents of phenolic acids and flavonoids in BH are significantly higher than in other berries like blueberries, mulberries, and goji berries, conferring stronger in vitro antioxidant and anti-inflammatory activities [36]. This indicates its greater potential compared to other berries in ameliorating HUA. This study systematically identified the primary chemical components of BHE as phenolic acids, flavonoids, anthocyanins, and iridoids using LC-MS/MS. Among these, chlorogenic acid and its derivatives were the main phenolic compounds, while glycosides of quercetin, kaempferol, and luteolin, such as rutin, hyperoside, and isoquercitrin, were the main flavonoids. These compounds have been proven to possess various biological functions, including inhibiting XOD activity, scavenging free radicals, and suppressing the release of inflammatory factors. Studies have confirmed that chlorogenic acid exhibits significant XOD inhibitory activity and antioxidant effects, directly blocking UA production [37]. Flavonoid glycosides like rutin and hyperoside can alleviate oxidative stress and inflammatory damage induced by HUA through scavenging reactive oxygen species and inhibiting the release of inflammatory factors [38,39]. The coexistence of these active components in BH suggests that, compared to single compounds, BHE may achieve better HUA amelioration effects through the regulation of multiple targets and signaling pathways. One limitation of this study is that we did not determine which specific compounds in BHE are primarily responsible for alleviating HUA. This issue will be addressed in our future research. Although several constituents detected in BHE have previously been reported to inhibit XOD or attenuate oxidative and inflammatory responses, their presence in BHE does not establish their individual contributions to the observed activity. The XOD inhibitory assay in the present study was performed on the whole extract, and neither fraction-specific nor compound-specific IC_50_ values were determined. Consequently, the active constituents and possible interactions among them remain unresolved. Bioactivity-guided fractionation followed by quantitative LC-MS analysis and XOD inhibition assays of purified compounds and defined mixtures will be required to identify the principal active constituents.

After ingestion, food undergoes sequential digestion in the mouth, stomach, and intestines. Bioactive components may be degraded or structurally transformed under the influence of gastric acid, digestive enzymes, and gut microbiota, leading to significant differences between actual in vivo activity and in vitro test results [40]. Previous studies have reported that the in vitro activity of most plant extracts is significantly higher than their in vivo activity, mainly due to the degradation of active components during digestion [41]. This study used an in vitro digestion model to investigate the impact of the digestive process on the main components and biological activities of BHE. The results showed that the total phenolic, total flavonoid, and total anthocyanin contents in BHE decreased significantly after digestion, with anthocyanins showing the most pronounced decline. This may be attributed to the susceptibility of their glycosidic bonds to hydrolysis by digestive enzymes and the ease of oxidation of phenolic hydroxyl groups [42]. Regarding biological activity, the XOD inhibitory activity and DPPH and ABTS radical scavenging capacities of BHE post-digestion were significantly lower than those of the undigested group. Notably, BHE at concentrations of 10 mg/mL and above still retained over 70% of its total phenolic and total flavonoid contents, XOD inhibitory activity, and radical scavenging capacity after digestion. Some studies have demonstrated declines in the active component content and biological activity of extracts from blueberries, mulberries, and other berries following in vitro digestion, which is highly consistent with the digestive variation pattern of BHE observed in this study [43]. Compared to extracts from common berries such as blueberries and mulberries, BHE exhibits superior retention of phenolics, flavonoids, and antioxidant activity during the digestive process. Nevertheless, as an extract, it remains challenging for BHE to completely avoid structural and functional damage by the digestive environment. In subsequent research, appropriate embedding materials will be considered for the protective encapsulation of BHE to construct a targeted delivery system, aiming to mitigate the degradation of active substances during digestion and enhance in vivo bioavailability. This study identified the optimal intake concentration of BHE as ≥10 mg/mL, providing direct experimental evidence for subsequent in vivo experiments and a reference for dosage in practical production and intake. The concentration of ≥10 mg/mL identified in the simulated digestion experiment should not be interpreted as a recommended human intake, because it represents an in vitro exposure concentration rather than an oral dose. Although the animal findings provide preliminary evidence of biological activity, differences in gastrointestinal exposure, absorption, metabolism, bioavailability, and extract composition preclude direct extrapolation to humans. Human dose-ranging and safety studies are therefore required before an effective intake level can be proposed.

This study established a HUA mouse model to investigate the ameliorative effects of BHE. HUA mice exhibited weight loss, reduced average daily food intake, elevated serum UA levels, decreased activities of antioxidant enzymes (SOD and CAT), and increased MDA content and expression of pro-inflammatory factors (IL-6, IL-1β, and TNF-α). These changes align with the pathological characteristics observed in clinical HUA patients [44]. Following BHE intervention, the aforementioned indices were reversed in a good dose-dependent manner. More importantly, the BHEH group showed effects comparable to or even better than those of the PC group. This phenomenon suggests that the UA-lowering mechanism of BHE may involve multiple pathways, including antioxidant and anti-inflammatory effects, reflecting the characteristic multi-pathway intervention of BHE as a food-derived active substance. However, we acknowledge that while this study provides valuable insights into the potential therapeutic role of BHE in HUA, our findings require further validation through rigorous experimentation in diverse animal models and human-relevant systems before any clinical implications can be considered.

The liver and kidneys are core organs for UA metabolism. The liver is the primary site for UA synthesis, where XOD serves as the key rate-limiting enzyme catalyzing the conversion of hypoxanthine and xanthine to UA [45]. The kidneys represent the main route for UA excretion, accomplished through glomerular filtration, reabsorption, and secretion [46]. Chronic high UA load can lead to liver and kidney dysfunction, which in turn exacerbates UA metabolism disorders, creating a vicious cycle [47]. This study found that serum and liver XOD activities were significantly elevated in HUA mice, accompanied by severe pathological damage to the liver and kidneys, and increased serum levels of ALT, AST, UREA, and CREA. These results suggest that HUA activates XOD activity, promotes UA synthesis, and leads to the deposition of urate crystals, damaging renal tubular epithelial cells, reducing renal excretory function, and causing abnormalities in liver and kidney function. BHE intervention significantly reduced XOD activity in both serum and liver, inhibiting UA production at its source. Concurrently, serum ALT and AST levels decreased, and liver pathological damage was alleviated. BHE also significantly ameliorated renal edema, lowered the kidney index and serum UREA and CREA levels, alleviated renal pathological damage, and restored renal excretory function. These findings indicate that BHE intervenes in UA production at the source while simultaneously mitigating liver and kidney damage and improving their function.

The balance of UA excretion is jointly regulated by the kidneys and the intestines [48]. Urate transporters constitute the core molecular mechanism of UA excretion. In the kidneys, ABCG2 is a key urate transporter mediating UA excretion, while URAT1 and GLUT9 are the main urate transporters mediating UA reabsorption [49]. Under normal conditions, UA excretion and reabsorption maintain a dynamic equilibrium. In the HUA state, renal ABCG2 expression is down-regulated, whereas URAT1 and GLUT9 expression is up-regulated, leading to decreased renal UA excretion and increased reabsorption, resulting in UA accumulation in the body [50]. The intestines serve as a secondary route for UA excretion, primarily mediated by ABCG2 [30]. Intestinal barrier integrity is fundamental for maintaining normal intestinal physiological function [51]. Down-regulation of the intestinal tight junction proteins ZO-1 and Occludin can lead to intestinal barrier damage, reduce intestinal ABCG2 expression, inhibit intestinal UA excretion, and even induce gut microbiota dysbiosis [52]. This study found that BHE could simultaneously regulate urate transporters in both the kidneys and intestines, promoting UA excretion through dual renal and intestinal pathways. In the kidneys, BHE restored ABCG2 expression and down-regulated URAT1 and GLUT9 expression, reestablishing the dynamic balance between renal UA excretion and reabsorption. In the intestines, BHE significantly restored the expression of tight junction proteins ZO-1 and Occludin, repaired intestinal barrier damage, and up-regulated intestinal ABCG2 expression to enhance intestinal UA excretion. Natural products often exert a single regulatory effect, either on hepatic UA production or renal urate transporters, resulting in limited improvement. In this study, BHE forms a synergistic liver–kidney–gut regulatory network by inhibiting hepatic UA production, restoring the balance of renal UA excretion and reabsorption, repairing the intestinal barrier, and enhancing intestinal UA excretion, thereby ameliorating HUA more efficiently and comprehensively. High UA exposure has been reported to promote tubulointerstitial inflammation through TLR4-related signaling, providing a possible molecular link between UA overload and renal inflammatory injury. In the present study, the increases in IL-6, IL-1β, and TNF-α and the renal inflammatory infiltration observed in HUA mice were attenuated by BHE. These findings are consistent with suppression of UA-associated inflammation. However, because TLR4 and its downstream signaling components were not examined, it remains unknown whether the anti-inflammatory effect of BHE was mediated through this pathway. Future studies should evaluate TLR4-related signaling to clarify this possibility [53,54].

Structural and functional dysregulation of the gut microbiota is a significant factor in the onset and progression of HUA [55]. The gut microbiota may influence UA metabolism through various means, including metabolizing purine precursors, maintaining intestinal barrier integrity, and influencing the expression of urate transporters [56]. Gut microbiota alterations have been reported in HUA patients and animal models, and these changes can correlate with UA levels [57]. 16S rRNA sequencing analysis in this study revealed that high UA load significantly reduced the richness and diversity of the gut microbiota in mice. At the phylum level, an imbalance in the Bacillota/Bacteroidota ratio was observed, with an increase in the abundance of harmful phyla like Fusobacteriota and a decrease in beneficial phyla like Actinomycetota. At the genus level, the abundance of harmful bacteria such as *Desulfovibrio* significantly increased, while beneficial bacteria like *Lachnospiraceae* decreased, consistent with the gut microbiota characteristics of HUA mice reported in previous studies [20]. Following BHE intervention, microbial diversity was significantly restored, and the community structure tended to resemble that of the CON group. BHE restored the balance of the Bacillota/Bacteroidota ratio, increased the abundance of beneficial bacteria including *Akkermansia* and *Lactobacillus*, and reduced the abundance of harmful bacteria such as *Desulfovibrio*. *Akkermansia*, a mucin-degrading bacterium, improves intestinal permeability by promoting mucus secretion and tight junction protein expression [58]. Reduced *Akkermansia* abundance is closely associated with intestinal barrier damage and impaired UA excretion in HUA patients [59]. In our study, increased *Akkermansia* in BHE intervention mice coincided with increased ZO-1 and Occludin expression. Lactobacillus has been reported to influence purine-related metabolism in some models, which could be relevant to UA homeostasis [60]. *Desulfovibrio* is a pro-inflammatory genus in the gut and its increased abundance can produce large amounts of endotoxins, disrupt the intestinal barrier, and trigger systemic inflammation [61]. In our dataset, *Desulfovibrio* decreased with BHE intervention, alongside improvements in inflammation-related indices. These microbial changes strongly suggest that BHE repairs the intestinal barrier and improves intestinal mucosal pathology via gut microbiota modulation. KEGG prediction further indicated that BHE reshapes microbial metabolic functions, including carbohydrate and amino acid metabolism and membrane transport, in ways favorable to host metabolic health.

In the present study, serum untargeted metabolomics revealed significant metabolic differences between HUA and normal mice, whereas the metabolic profile of BHE-treated mice shifted markedly toward that of the normal mice. Identification of differential metabolites and KEGG enrichment analysis indicated that the metabolites modulated by BHE were primarily enriched in purine metabolism, biosynthesis of secondary metabolites, microbial metabolism and other pathways. Notably, metabolites related to UA metabolism, such as uracil and allantoin, were significantly increased in the HUA group but were significantly reversed following BHE intervention. Correlation analysis integrating the microbiome and metabolome further revealed that the relative abundances of Akkermansia and Lactobacillus were significantly negatively correlated with uracil and UA levels. These results confirm that BHE regulates UA metabolism disorders by improving the gut microbiota and its associated metabolic functions. A critical direction for future research is to establish the causal link between the gut microbiota and host UA metabolism through fecal microbiota transplantation experiments. Furthermore, integrating germ-free mouse models with controlled microbial colonization strategies would help clarify whether the therapeutic effects of BHE on HUA are dependent on specific microbial communities or their derived metabolites.

## 5. Conclusions

In conclusion, this study systematically investigated the ameliorative effects and molecular mechanisms of BHE on HUA mice through multiple dimensions including LC-MS/MS analysis, in vitro digestion, and in vivo animal experiments. These findings indicate that BHE reduced serum UA in HUA mice, and this effect was associated with lower XOD activity, regulation of renal and intestinal urate transporters, improved intestinal barrier integrity, and changes in the gut microbiota and serum metabolic profile. The relative causal contributions of these processes require further investigation.

## Figures and Tables

**Table 1:** RT-qPCR Primers.

Primers	Primer Sequence	Gene Accession Number
*Gapdh*	F: AATGGATTTGGACGCATTGGT	NM_001289726.2
	R: TTTGCACTGGTACGTGTTGAT	
*Abcg2*	F: GAACTCCAGAGCCGTTAGGAC	NM_001355477.2
	R: CAGAATAGCATTAAGGCCAGGTT	
*Urat1*	F: TCTTCTGGCCGTCTCCATC	NM_009203.3
	R: AGCTGCCATTGAGGTTGTCG	
*Glut9*	F: CTTCCTCTACGGGTACAACCT	NM_001102414.1
	R: CAGAGCAGAGTCAGGGTATCC	
*Zo-1*	F: GCCGCTAAGAGCACAGCAA	NM_009386.3
	R: GCCCTCCTTTTAACACATCAGA	
*Occludin*	F: TGAAAGTCCACCTCCTTACAGA	NM_008756.2
	R: CCGGATAAAAAGAGTACGCTGG	

**Table 2:** Identification of main chemical components in BHE by LC-MS/MS.

No.	Retention Time (min)	Molecular Formula	Theoretical MS (*m*/*z*)	Measured *m*/*z* (ESI^+^/ESI^−^)	Compounds
1	13.380	C_16_H_18_O_9_	354.0951	355.1019/353.0885	Caffeoylquinic acid
2	13.380	C_16_H_18_O_9_	354.0951	355.1019/353.0885	Cryptochlorogenic acid
3	13.380	C_16_H_18_O_9_	354.0951	355.1019/353.0885	Chlorogenic acid
4	13.380	C_16_H_18_O_9_	354.0951	355.1019/353.0885	Neochlorogenic acid
5	13.615	C_21_H_22_O_12_	466.1111	467.1181/465.1043	Taxifolin-7-O-glucoside
6	16.097	C_27_H_30_O_16_	610.1534	611.1610/609.1449	Kaempferol-3,7-di-O-glucoside
7	16.097	C_27_H_30_O_16_	610.1534	611.1610/609.1449	Kaempferol-3-O-β-D-sophoroside
8	16.097	C_27_H_30_O_16_	610.1534	611.1610/609.1449	Luteolin-7,3′-di-O-glucoside
9	16.097	C_27_H_30_O_16_	610.1534	611.1610/609.1449	Quercetin 3-O-rutinoside
10	17.504	C_16_H_24_O_10_	376.1369	377.1441/375.1300	Loganic acid
11	17.504	C_16_H_24_O_10_	376.1369	394.1712/375.1300	8-Epiloganic acid
12	20.924	C_27_H_30_O_16_	610.1534	628.1878/655.1519	Kaempferol-3-O-gentiobioside
13	22.379	C_21_H_21_O_11_	449.1084	449.1086/447.093	Cyanidin-3-O-glucoside
14	23.035	C_21_H_22_O_12_	466.1111	467.1191/465.1049	Plantagoside
15	23.363	C_17_H_24_O_10_	388.1369	406.1707/433.1356	7-Ketologanin
16	24.488	C_17_H_26_O_10_	390.1526	408.1868/425.1233	Loganin
17	28.798	C_15_H1_2_O_8_	320.0532	321.0598/319.0466	Dihydromyricetin
18	33.385	C_27_H_30_O_16_	610.1534	611.1617/609.1470	Quercetin-3-galactosyl(1→2) rhamnose
19	33.385	C_27_H_30_O_16_	610.1534	611.1617/609.1470	Quercetin-3-O-neohesperidoside
20	33.385	C_27_H_30_O_16_	610.1534	611.1617/609.1470	Rutin
21	33.385	C_27_H_30_O_16_	610.1534	611.1617/609.1470	6-Hydroxykaempferol-3-O-rutinoside
22	33.385	C_27_H_30_O_16_	610.1534	611.1617/609.1470	Rhodiosin
23	33.385	C_27_H_30_O_16_	610.1534	611.1617/609.1470	Quercetin-3-O-(2′′-β-D-glucoside)-α-L-rhamnoside
24	33.760	C_21_H_20_O_12_	464.0955	465.1030/463.0884	6-Hydroxykaempferol-3-O-β-D-glucoside
25	33.760	C_21_H_20_O_12_	464.0955	465.1030/463.0884	Isoquercitrin
26	33.760	C_21_H_20_O_12_	464.0955	465.1030/463.0884	Delphinidin-3-O-galactoside
27	33.760	C_21_H_20_O_12_	465.1033	465.1030/463.0884	Quercetin-7-O-β-D-glucoside
28	33.760	C_21_H_20_O_12_	465.1033	465.1030/463.0884	6-Hydroxyluteolin glucoside
29	33.760	C_21_H_20_O_12_	465.1033	465.1030/463.0884	Hyperoside
30	35.353	C_25_H_24_O_12_	516.1268	517.1337/515.1201	Isochlorogenic acid C
31	35.353	C_25_H_24_O_12_	516.1268	517.1337/515.1201	Isochlorogenic acid B
32	35.353	C_25_H_24_O_12_	516.1268	517.1337/515.1201	1,4-Dicaffeoylquinic acid
33	35.353	C_25_H_24_O_12_	516.1268	517.1337/515.1201	1,5-Dicaffeoylquinic acid
34	35.353	C_25_H_24_O_12_	516.1268	517.1337/515.1201	3,5-Dicaffeoylquinic acid
35	35.353	C_25_H_24_O_12_	624.169	625.1763/623.1618	6-Methoxykaempferol-3-O-rutinoside
36	35.353	C_25_H_24_O_12_	624.169	625.1763/623.1618	Isorhamnetin-3-O-glucoside-7-O-rhamnoside
37	35.353	C_25_H_24_O_12_	624.169	625.1763/623.1618	Narcissoside
38	35.353	C_25_H_24_O_12_	624.169	625.1763/623.1618	Isorhamnetin-3-O-neohesperidoside

## Data Availability

The data presented in this study are available in [NCBI Sequence Read Archive (SRA)] at [https://www.ncbi.nlm.nih.gov/sra/PRJNA1506056], reference number [PRJNA1506056] (accessed on 3 August 2026).

## Abbreviations

The following abbreviations are used in this manuscript:

BHE	Blue honeysuckle extract
HUA	Hyperuricemia
UA	Uric acid
XOD	Xanthine oxidase
URAT1	Urate transporter 1
GLUT9	Glucose transporter
ABCG2	ATP binding cassette subfamily G member
ZO-1	Zonula occludens-1
DPPH	2,2-Diphenyl-1-picrylhydrazyl
ABTS	3-ethylbenzthiazoline-6-sulphonate
IL-6	Interleukin-6
IL-1β	Interleukin-1β
TNF-α	Tumor necrosis factor-α
ALT	Alanine aminotransferase
AST	Aspartate aminotransferase
H&E	Hematoxylin and eosin
GAPDH	Glyceraldehyde-3-phosphate dehydrogenase
